# Powerful Antioxidants and Cytotoxic Activities of the Methanol Extracts from Eight Soybean Cultivars

**DOI:** 10.3390/molecules27092895

**Published:** 2022-05-01

**Authors:** Mohamed A. Abd Elhamid, Abd Elrahman S. Mandour, Tarek A. Ismail, Ahmed M. Al-Zohairy, Sanaa Almowallad, Leena S. Alqahtani, Ali Osman

**Affiliations:** 1 Genetics Department, Faculty of Agriculture, Zagazig University, Zagazig 44511, Egypt; mohabdelsalames88@gmail.com (M.A.A.E.); mandourabdelrahman@gmail.com (A.E.S.M.); drtarek2023@gmail.com (T.A.I.); alzohairy@yahoo.com (A.M.A.-Z.); 2 Department of Biochemistry, Faculty of Sciences, University of Tabuk, Tabuk 71491, Saudi Arabia; salmowaled@ut.edu.sa; 3 Department of Biochemistry, Faculty of Science, University of Jeddah, Jeddah 23445, Saudi Arabia; lsalqahtani@uj.edu.sa; 4 Biochemistry Department, Faculty of Agriculture, Zagazig University, Zagazig 44511, Egypt

**Keywords:** soybean, DPPH assay, MTT assay, cytotoxicity, isoflavones, flavonoids

## Abstract

In the present study, the chemical composition and total phenolic (TPC) and total flavonoid contents (TFC) of eight soybean cultivars (Giza 21, Giza 22, Giza 35, Giza 111, Giza 82, Giza 83, Crawford, and Holliday) were estimated. Moreover, antioxidant activity and in vitro cytotoxic activities against HepG-2 and MCF-7 were evaluated. Giza 21, Giza 111, and Crawford cultivars recorded higher than 40% crude protein. The analysis revealed that TPC values in seed extracts ranged from 10.5 mg GAE/g extract in Giza 35 to 6.4 mg GAE/g extract in Giza 22. TFC varied from 1.20 mg QE/g extract in Giza 111 to 0.55 mg QE/g extract in Crawford. Giza 35 exhibited the highest content of genistein and daidzein and the highest free radical scavenging activity (61.833%). The results of the MTT assay demonstrated that the soybean methanolic extracts inhibited the proliferation of HepG-2 and MCF-7 cells in a dose-dependent manner. Giza 35 exhibited the highest cytotoxic activity. In conclusion, Giza 35 cultivar recorded the highest TPC and TFC values and antioxidant and cytotoxic activities. Therefore, this cultivar can be used as a source for the production of pharmaceutical and medicinal products rather than as a nutritional source of protein.

## 1. Introduction

Natural bioactive compounds isolated from different sources have protective activities against various diseases such as cancer [1]. Among these natural components, polyphenols have received special attention due to their potent antibacterial, antiviral, antioxidant activities and because they inhibit the proliferation of cancer cells [2]. Due to their biological activities, polyphenols have been extensively researched for decades. Consequently, polyphenols isolated from different agricultural crops have been investigated with the aim of supplying nutrition that has useful effects on human health [3].

Among these agricultural crops, legume seeds have received special attention due to their natural bioactive components, such as phenolics, flavonoids, and isoflavones [4].

Soybean (*Glycine max* (L.) Merr.) is one of the world’s most widely planted leguminous crops, growing in tropical, subtropical, and temperate climates and supplying plentiful protein (about 40%) and oil (about 20%) for human and animal consumption [5]. Many of the phenolic compounds present in plant tissues have antioxidant properties. All phenolics have reactive oxygen species (ROS) scavenger capabilities. Most phenolic compounds are concentrated in the seed of the legumes [6]. The bioactive phenolic compounds present in grain legumes make them suitable candidates for creating new functional foods [7]. These compounds might offer indirect protection by activating endogenous defense systems and by the modulation of cellular signaling processes. The change in the bioactivity of phenolic compounds exemplifies their importance in food products. They have many health benefits, such as anticarcinogenic, antioxidant, and antimicrobial properties [8,9]. Except for isoflavonoids, limited research has been conducted on the other phenolic classes present in soybean.

The local soybean varieties have a wide range of maturity and diverse morphology. Apart from these, they are high-yielding with good desirable agronomic characteristics under intercropping conditions. Thus, this study aims to examine the chemical composition of 6 local soybean cultivars (Giza 21, 22, 35, 82, 83, and 111) compared with American cultivars of soybean such as Crawford and Holliday. On the other hand, seed phenolic-rich extracts of different eight soybean cultivars as antioxidant and cytotoxic agents were estimated.

## 2. Results

### 2.1. Proximate Analysis

Descriptive values of eight soybean cultivars for proximate analysis (crude protein, ash, fat, crude fiber, carbohydrate, and moisture contents), total phenolic (TPC), and total flavonoid contents (TFC) are presented in Table 1. Crude protein values ranged between 35.17% in Giza 35 and 44.23% in Giza 111. Cultivars recorded higher than 40% crude protein were Giza 21, Giza 111, and Crawford. Moisture content ranged from 3.57% in Holliday to 5.20% in Giza 111. Ash content in eight soybean cultivars ranged from 4.05% in Giza 21 to 5.16% in Holliday. Total fat also varied from 20.97% in Giza 35 to 18.15% in Giza 82. The carbohydrate content ranged from 27.70% in Holliday to 20.49% in Giza 111. The crude fiber content varied from 10.85% in Giza 35 to 6.14% in Crawford. The results revealed nonsignificant differences among some cultivars and significant differences among others in constituents, reflecting the variations in genetic background and/or origin.

### 2.2. Total Phenolic (TPC) and Total Flavonoid Content (TFC)

TPC (mg GAE/g extract) and TFC (mg QE/g extract) of extracts from soybean cultivars are presented in Table 2. A wide variation was observed for the TPC and TFC and the cultivars differed significantly with respect to these parameters. The analysis revealed that TPC values in seed extracts range from 10.5 mg GAE/g extract in Giza 35 to 6.4 mg GAE/g extract in Giza 22. TFC varied from 1.20 mg QE/g extract in Giza 111 to 0.55 mg QE/g extract in Crawford.

### 2.3. Phenolic, Flavonoid, and Isoflavone Compounds Identification by HPLC

**Figure 1:**
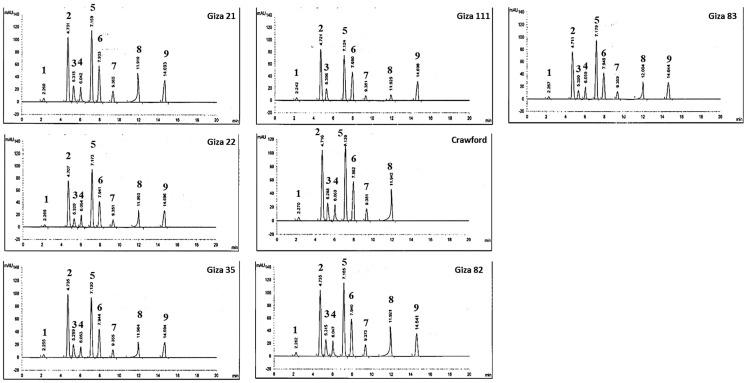
HPLC of major phenolic, flavonoid, and isoflavone compounds (1: syringic, 2: quercetin, 3: gallic acid, 4: benzoic acid, 5: genistein, 6: daidzein, 7: *p*-coumaric, 8: glycitein, and 9: ferulic acid) in eight soybean cultivar extracts.

A representative chromatogram of the HPLC phenolic compounds analysis of eight soybean cultivars extracts is shown in Figure 1. Nine peaks (syringic, quercetin, gallic acid, benzoic acid, genistein, daidzein, *p*-coumaric, glycitein, and ferulic acid) dominated in the chromatograms from the separation of Giza 21, Giza 22, Giza 35, Giza 82, Giza 83, and Holliday extracts. On the other hand, benzoic acid and ferulic acid were absent on chromatograms from the separation of Giza 111 and Crawford, respectively. Table 3 summarizes the contents of phenolic compounds 1–9 in the eight soybean cultivars extracts. It was shown that quercetin, genistein, daidzein, and glycitein were the main phenolic compounds in the eight soybean cultivar extracts. The main flavonoid compound of the eight soybean cultivars was quercetin. Giza 111 exhibited the highest content of quercetin (37.34%), followed by Giza 35 (30.80%), whereas Giza 83 recorded the lowest quercetin amount (26.05%). An HPLC chromatogram shows the isoflavone compounds (genistein, daidzein, and glycitein). Giza 35 exhibited the highest content of genistein and daidzein (30.00% and 15.07%, respectively), whereas Giza 111 recorded the lowest genistein and daidzein values (15.87% and 1.87%, respectively). The main phenolic compound of the eight soybean cultivars was gallic acid. The highest gallic acid content was recorded in the case of Giza 35 (7.79%), followed by Giza 111 and Giza 83 (7.53% and 7.50%, respectively).

### 2.4. Antioxidant Activity

The antioxidant activities of the eight soybean cultivars are shown in Table 4. Giza 35 exhibited the highest free radical scavenging activity (61.833%), whereas Giza 82 had the lowest (47.928%). There were significant differences (*p* < 0.001) in the free radical scavenging activities of the Giza 35 and others cultivars (data not shown). The extracts from all soybean cultivars demonstrated the capability to scavenge DPPH free radicals. Overall, antioxidant activity increased proportionally to the increased concentration, and a linear relationship between DPPH radical scavenging activity and concentration was established (data not shown). The results of the IC_50_ value of the soybean cultivar methanolic extract are presented in Table 5. Giza 35, Giza 111, Giza 83, Giza 21, and Crawford methanol extracts had stronger antioxidant activities than TBHQ. The required concentrations for scavenging of 50% radicals were 45, 52, 55, 57, 61, and 62 mg/mL for Giza 35, Giza 111, Giza 83, Giza 21, Crawford, and TBHQ, respectively.

Table 5 presents the antioxidant activity of soybean cultivars methanolic extract at 800 µg/mL, determined as FRAP. FRAP test showed significant differences between Giza 35 and other soybean cultivars. FRAP values ranged from 770 to 1900 µmol Fe^+2^/g extract. FRAP was the highest for Giza 35 (1900 µmol Fe^+2^/g extract), followed by those of Giza 83 (1600 µmol Fe^+2^/g extract) and Giza 111 (1400 µmol Fe^+2^/g extract). No significant differences were observed (*p* < 0.001) between Giza 111 and Giza 83. FRAP was the lowest for Holliday (770 µmol Fe^+2^/g extract), with no significant differences (*p* < 0.001) between Giza 21, Giza 22, and Holliday.

### 2.5. Cytotoxic Activity

**Figure 2:**
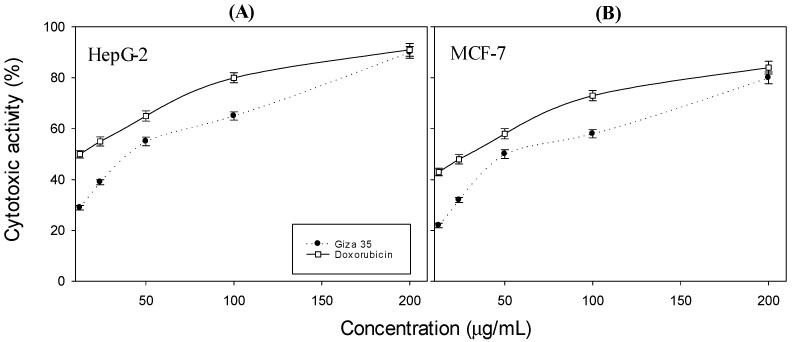
Cytotoxic activity of methanolic extracted from soybean Giza 35 at different concentrations (12.5, 25, 50, 100, and 200 µg/mL) against HepG-2 (**A**) and MCF-7 (**B**) compared to that of Doxorubicin as a control. All data are expressed as the mean (*n* = 3) with ±SD.

Soybean methanolic extracts at different concentrations (12.5, 25, 50, 100, and 200 µg/mL) from eight soybean cultivars were tested in vitro for their cytotoxic activities on the HepG-2 and MCF-7 human cancer cell lines using the MTT assay. The percentages of the viable cells and their cytotoxic activity were measured and compared with the control, Doxorubicin (Table 6 and Table 7). Overall, cell viability (%) decreased with the increase in concentration, and a linear relationship between cell viability and concentration was established. Cytotoxic activity of methanolic extracted from soybean Giza 35 (the highest cytotoxic activity) at different concentrations (12.5, 25, 50, 100, and 200 µg/mL) against HepG-2 (A) and MCF-7 (B) was compared to that of Doxorubicin as a control (Figure 2). The results of the MTT assay demonstrated that the soybean methanolic extracts inhibited the proliferation of HepG-2 and MCF-7 cells in a dose-dependent manner.

## 3. Discussion

The main objectives of the current study were to investigate the chemical composition of eight soybean cultivars (Giza 21, Giza 22, Giza 35, Giza 111, Giza 82, Giza 83, Crawford, and Holliday), TPC, TFC, antioxidant activity and cytotoxic activity against human cell lines (HepG-2 and MCF-7). The results of chemical composition revealed no significant differences among some cultivars and significant differences among others in constituents, reflecting the variations in genetic background and/or origin. The values observed in the present study were similar to those obtained by other researchers [10,11].

In the present study, methanolic extracts from eight soybean cultivars were prepared and analyzed for TPC and TFC. Giza 35 exhibited the highest TPC and TFC and recorded the highest genistein, daidzein, and quercetin contents. Overall, when TPC and TFC are increased in soybean samples, the antioxidant activity increases, as indicated by the results of DPPH and FRAP. The antioxidant activity of the phenolic-rich cultivar (Giza 35) showed high levels of DPPH radical scavenging activity. Furthermore, the values observed in the present study were in line with those obtained by other researchers [12,13,14].

The Folin–Ciocalteu method responds differently to different phenolic chemicals in food matrix samples depending on the number of phenolic groups they contain [15]. Because of the phenolic moiety’s reactivity, some polyphenolic compounds have been found to have antioxidant properties, scavenging free radicals via electron donation or hydrogen donation [16]. Isoflavones make up the majority of TPC in soybean seeds. According to several studies, TPC and total isoflavone concentration are favorably associated [17,18,19]. In the present work, HPLC was used to identify phenolic compounds. Nine peaks (syringic, quercetin, gallic acid, benzoic acid, genistein, daidzein, *p*-coumaric acid, glycitein, and ferulic acid) dominated different soybean cultivars. Previous studies have reported that soybean contains several phenolic acids, such as syringic, ferulic, sinapic, *p*-coumaric, hydroxybenzoic, caffeic, and chlorogenic acids, and those total phenolic compounds are highly positively correlated with hydroxybenzoic acids, such as gentisic acid and salicylic acid [20].

In comparison to traditional medicines, dietary phytochemicals have been shown to prevent disease start or progression with far less damage and cost [21]. Phytochemicals’ efficiency in the therapy of many types of cancer has been extensively studied [22,23,24]. On this therapeutic basis, natural compounds regulate different cellular pathways and enzyme activities that are often altered in cancer cells.

In the current work, soybean methanolic extracts from eight soybean cultivars were tested in vitro for their cytotoxic activities compared with the control, Doxorubicin, on the HepG-2 and MCF-7 human cancer cell lines using the MTT assay. The results of the MTT assay demonstrated that the soybean methanolic extracts inhibited the proliferation of HepG-2 and MCF-7 cells in a dose-dependent manner. Giza 35 recorded the highest cytotoxic activity compared to other cultivars and exhibited the highest TPC and TFC. Furthermore, it recorded the highest genistein, daidzein, and quercetin contents. Overall, the trend in cytotoxic activity was similar to that of TPC, TFC, and isoflavones content. Flavonoids represent an important group of bioactive compounds derived from soybean with known biological activity in cells. From the modulation of inflammation to the inhibition of cell proliferation, flavonoids have been described as important therapeutic adjuvants against several diseases, including diabetes, arteriosclerosis, neurological disorders, and cancer [25]. Various studies have suggested different mechanisms for anticancer action of phenolic compounds or polyphenol-rich extracts. Insulin-like growth factor (IGF), known as a potential biomarker, plays a key role in the development of many cancers, including breast, lung, and colorectal cancers. It is thought that the high activity of these multifunctional peptides (IGFs) plays a meaningful role in all stages of carcinogenesis. Therefore, finding potential inhibitors of IGF should be a primary focus of research. Polyphenols might be promising inhibitors, as they do not have side effects when used in chemoprevention and cancer therapy [26]. Polyphenols exhibit cytotoxicity effects against numerous kinds of malignancies, including colorectal, multiple myeloma, breast, pancreatic, prostate, oral, and lung cancers, mainly described in previously published literature. The cytotoxicity effect of polyphenolic compounds may be related to induced apoptosis by cell cycle arrest at the G2/M phase and rapid accumulation in the cell [27]. Plant polyphenols can prevent cancer development by modulating some of the signal transduction pathways related to the cancer process. The main anticancer mechanisms include modulation of proinflammatory cytokines and modulation of several apoptotic proteins like NF-ĸB, cyclooxygenase-2, STAT3, and endothelin-1 [28].

## 4. Materials and Methods

### 4.1. Chemicals

2,2-Diphenyl-1-picrylhydrazyl (DPPH), 2,4,6-tri(2-pyridyl)-S-triazine (TPTZ), Folin–Ciocalteu’s phenol reagent (FCR), gallic acid, and quercetin were obtained from the Sigma-Aldrich Chemical Company (St. Louis, MO, USA). Methanol and glacial acetic acid were obtained from Nasr Company for Chemical Industries, Cairo, Egypt. All other chemicals used in experiments were of an analytical grade.

### 4.2. Plant Materials

Eight cultivars of soybean seeds (*Glycine max* L.) were used in this investigation: Giza 21, Giza 22, Giza 35, Giza 111, Crawford, Giza 82, Giza 83, and Holliday (Table 8). Seed samples were obtained from the Leguminous Crops Department Research (LCDR), Field Crops Research Institute, Agricultural Research Center, Ministry of Agriculture, Giza, Egypt.

### 4.3. Proximate Analysis

Soybean samples were analyzed in triplicate for crude proteins, moisture, total ash, fat, crude fiber, and carbohydrate using the procedures given in the AOAC [29]. All proximate values were provided in g/100 g dry weight [30].

### 4.4. Phenolic Compounds Extraction and Characterization

Seeds from eight cultivars of soybeans were ground and defatted for 6–8 h using a Soxhlet apparatus with hexane. Phenolic compounds were extracted from the defatted raw seeds with 70% (*v*/*v*) aqueous methanol at a solid-to-solvent ratio of 1:10 (*w*/*v*) at 40 °C min in a Soxhlet apparatus. The extracts were filtered through a filter paper and concentrated in a rotary evaporator (Büchi rotary evaporator, Flawil, Switzerland) below 40 °C. The resultant aqueous solutions were frozen and lyophilized [31].

### 4.5. Total Phenolics Content (TPCs) Estimation

Folin–Ciocalteu assay was used to estimate TPC in the soybean seeds’ methanolic extracts (2000 µg/mL), as described by Singleton et al. [15] Gallic acid (10–2000 µg/mL) was used to obtain the following calibration equation: y = 0.001x + 0.0563; R_2_ = 0.9792, where y and x are the gallic acid absorbance and concentration in µg/mL, respectively. The absorbance was read at 725 nm using a spectrophotometer (Jenway, 6405 UV/Vis, Chelmsford, UK).

### 4.6. Total Flavonoid Content (TFC) Estimation

TFC in the soybean seeds’ methanolic extracts (2000 µg/mL) was estimated according to the protocol of Ordonez et al. [32] as described in Abdel-Shafi et al. [8] Quercetin (10–1000 µg/mL) was used to obtain the following calibration equation: y = 0.0012x + 0.008; R_2_ = 0.944, where y and x are the quercetin absorbance and concentration in µg/mL, respectively. The absorbance of color was recorded at 420 nm by using a spectrophotometer (Jenway, 6405 UV/Vis, Chelmsford, UK).

### 4.7. Polyphenolic Compounds Identification

High-Performance Liquid Chromatography (HPLC) was used to identify polyphenolic compounds in the methanolic extracts of eight soybean seeds cultivars. HPLC-Agilent 1100 apparatus is composed of a C18 column (125 mm × 4.60 mm, 5 µm particle size), two LC pumps, and a UV/Vis detector. The phenolic acid conditions used were as follows: column temperature, 30 °C; injection volume, 20 µL; wavelength, 280 nm; total HPLC run time, 50 min; a mobile phase solution A of 1% aqueous solution acetic acid and solution B of acetonitrile (34, 35). The mobile phase was programmed as follows: 0–5 min, solution B at 5–15%; 5–35 min, solution B at 15–35%; 35–40 min, solution B at 35–45%; 40–50 min, solution B at 45–5%. The mobile phase was pumped at a constant flow rate of 1.0 mL/min. The isoflavone conditions were as follows: column temperature, 20 °C; injection volume, 20 µL; wavelength, 260 nm; total HPLC run time, 50 min; mobile phase solution A of 0.1% aqueous solution acetic acid and solution B of acetonitrile. The mobile phase was identical to the one used for the method for the phenolic acids. Before analysis, all samples were filtered through a 0.45 µm membrane filter (Millipore, Billerica, MA, USA). Samples were quantified by comparing the retention times with known authentic standards. All measurements were done in triplicate.

### 4.8. Antioxidant Capacity (DPPH Assay)

The scavenging effect of phenolic compounds from the methanolic extracts of eight soybean cultivars was measured according to Ramadan et al. [33] Briefly, 500 µL of a methanolic solution of phenolic extract at different concentrations (25, 50. 100, 200, 400, and 800 µg/mL) was mixed with 2 mL of a freshly prepared methanolic solution of DPPH^•^ (1 mM), Sigma-Aldrich Chemical Company (St. Louis, MO, USA). Then, 1 mM methanolic solution of DPPH^•^ without any addition was used as a control. The mixture was vortexed and incubated at room temperature for 30 min. The absorbance was read at 517 nm using a spectrophotometer. The *antioxidant activity* was calculated from the following Equation:

Antioxidant activity (%)=(Ab control−Ab sample)/ Ab control×100.

### 4.9. FRAP-Reducing Antioxidant Power

The FRAP reagent was prepared using a previously described method [34]. The stock solutions included 300 mM acetate buffer (3.1 g C_2_H_3_NaO_2_·3H_2_O and 16 mL C_2_H_4_O_2_), pH 3.6, 10 mM TPTZ (2, 4, 6-tripyridyl-s-triazine) the solution in 40 mM HCl, and 20 mM FeCl_3_·6H_2_O solution. The fresh working solution was prepared by mixing 25 mL acetate buffer, 2.5 mL TPTZ solution, and 2.5 mL FeCl_3_·6H_2_O solution and was then warmed at 37 °C before usage. For the assay, 1 mL of extract solution was mixed with 1 mL of working FRAP reagent. Then, the mixture was kept at room temperature for 30 min. The absorbance of the reaction mixture was measured at 593 nm using a spectrophotometer. FRAP results were expressed as µmol Fe^2+^ equivalents per g of extract using the calibration curve for FeSO_4_.

### 4.10. The Effect of Soybean Extracts on Cancer Cell Viability In Vitro (MTT Assay)

HepG2 and MCF7 cells were grown in Dulbecco’s modified Eagle’s medium (DMEM, Sigma-Aldrich, Burlington, MA, USA) supplemented with 10% heat-inactivated fetal bovine serum (FBS), penicillin (10 U/mL, Sigma-Aldrich, Burlington, MA, USA), and streptomycin (10 g/mL, Sigma-Aldrich, Burlington, MA, USA). The cultures were incubated in the presence of 5% CO_2_ at 37 °C and 100% relative humidity.

The cells were seeded in 96-well microplates at a density of 10 × 10^3^ cells/well and were grown for 24 h at 37 °C in 5% CO_2_ before the addition of the samples. The cells were treated with various concentrations (12.5, 25, 50, 100, and 200 µg/mL) of soybean cultivars methanolic extracts dissolved in phosphate-buffered saline (PBS). Cell viabilities were determined after 48 h incubation using the colorimetric MTT assay (Promega, Madison, WI, USA) [35]. The cell viability (%) was estimated based on the levels of formazan production, according to the absorbance at 550 nm. Triton X-100 (10 µL of a 10% solution) was used as the positive control, whereas untreated cells (0 µg/mL vehicle only) were used as the negative control. The percentage of *cell viability* was calculated by the following formula:

cell viability (%)=(Ab sample/Ab control)×100

*Cytotoxic activity* (%) of soybean extracts was calculated using the following formula:

Cytotoxic activity (%)=100 %−cell viability (%)

### 4.11. Statistical Analysis

The data of all studied characteristics of all soybean cultivars were statistically analyzed. In every trial, the order of samples was randomized and performed in three replicates per cultivar. Moreover, the experimental trials were conducted on different days. Analysis of variance (ANOVA) was performed for a completely randomized design on all data. The differences among cultivars, concentrations, and their interactions were determined by Tukey’s range test (*p*  ≤  0.05).

## 5. Conclusions

Giza 35 exhibited the highest cytotoxic activity. In conclusion, Giza 35 cultivar recorded the highest TPC, TFC, antioxidant activity, and cytotoxic activity. Therefore, this cultivar can be used as a source for the production of pharmaceutical and medicinal products rather than as a nutritional source of protein.

## Figures and Tables

**Table 1:** Chemical composition of the eight soybean cultivars.

Cultivar	Constituents of the Sample (%)					
	Proteins	Carbohydrates	Fat	Ash	Crud Fibre	Moisture
Giza 21	43.67 ± 2.00 ^ab^	21.81 ± 1.00 ^cd^	19.42 ± 0.89 ^ab^	4.05 ± 0.19 ^c^	6.70 ± 0.31 ^de^	4.35 ± 0.20 ^cde^
Giza 22	37.48 ± 1.72 ^c^	26.72 ± 1.22 ^ab^	19.53 ± 0.90 ^ab^	4.50 ± 0.20 ^bc^	6.95 ± 0.32 ^cde^	4.82 ± 0.27 ^bcd^
Giza 35	35.17 ± 1.61 ^c^	23.96 ± 1.09 ^bc^	20.97 ± 0.96 ^a^	4.00 ± 0.18 ^c^	10.85 ± 0.40 ^a^	5.05 ± 0.23 ^ab^
Giza 111	44.23 ± 2.03 ^a^	20.49 ± 0.94 ^d^	19.13 ± 0.88 ^ab^	4.25 ± 0.19 ^c^	6.70 ± 0.31 ^de^	5.20 ± 0.24 ^a^
Crawford	42.83 ± 1.97 ^ab^	21.99 ± 1.00 ^cd^	20.03 ± 0.92 ^ab^	4.17 ± 0.19 ^c^	6.14 ± 0.28 ^e^	4.84 ± 0.22 ^abc^
Giza 82	39.80 ± 1.83 ^abc^	25.48 ± 1.17 ^ab^	18.15 ± 0.83 ^b^	4.90 ± 0.22 ^ab^	7.77 ± 0.36 ^bc^	3.90 ± 0.18 ^def^
Giza 83	38.85 ± 1.78 ^bc^	26.95 ± 1.23 ^ab^	18.96 ± 0.87 ^ab^	4.32 ± 0.20 ^bc^	7.17 ± 0.33 ^bcd^	3.75 ± 0.17 ^ef^
Holliday	37.05 ± 1.70 ^c^	27.70 ± 1.27 ^a^	18.40 ± 0.85 ^b^	5.16 ± 0.23 ^a^	7.94 ± 0.36 ^b^	3.57 ± 0.16 ^f^
*p*-value	<0.001	<0.001	0.029	<0.001	<0.001	<0.001

All data are expressed as the mean (*n* = 3) with ±SD in the same column; the a–f letters indicate significant differences between means (*p* < 0.01).

**Table 2:** Total phenolic (TPC) and total flavonoid contents (TFC) in eight soybean cultivars extracts.

Cultivar	TF mg QE/g Extract	TPC mg GAE/g Extract
Giza 21	1.10 ± 0.05 ^ab^	7.90 ± 0.29 ^d^
Giza 22	0.75 ± 0.02 ^c^	6.40 ± 0.30 ^e^
Giza 35	1.05 ± 0.05 ^b^	10.50 ± 0.48 ^a^
Giza 111	1.20 ± 0.06 ^a^	9.40 ± 0.43 ^ab^
Crawford	0.55 ± 0.03 ^e^	8.09 ± 0.37 ^cd^
Giza 82	0.65 ± 0.03 ^de^	9.02 ± 0.42 ^bc^
Giza 83	0.56 ± 0.03 ^e^	10.25 ± 0.47 ^a^
Holliday	0.71 ± 0.03 ^cd^	7.20 ± 0.33 ^de^
*p*-value	<0.001	<0.001

All data are expressed as the mean (*n* = 3) with ±SD in the same column; the a, b, c, d and e letters indicate significant differences between means (*p* < 0.01).

**Table 3:** Major phenolic, flavonoid, and isoflavone compounds in the eight soybean cultivar extracts estimated by HPLC.

Cultivar	Concentration (%)								
	Syringic Acid	Quercetin	Gallic Acid	Benzoic Acid	Genistein	Daidzein	*p*-Coumaric Acid	Glycitein	Ferulic Acid
Giza 21	0.93 ± 0.06 ^d^	30.05 ± 1.38 ^bc^	6.61 ± 0.23 ^b^	5.25 ± 0.19 ^b^	25.74 ± 0.89 ^e^	12.82 ± 0.44 ^c^	2.82 ± 0.15 ^c^	10.24 ± 0.18 ^b^	5.54 ± 0.15 ^d^
Giza 22	1.31 ± 0.06 ^a^	29.25 ± 1.34 ^bc^	6.00 ± 0.21 ^c^	4.94 ± 0.17 ^c^	27.72 ± 0.48 ^cd^	13.11 ± 0.23 ^c^	3.60 ± 0.17 ^b^	7.10 ± 0.33 ^e^	6.97 ± 0.12 ^c^
Giza 35	0.50 ± 0.03 ^e^	30.80 ± 1.07 ^b^	7.79 ± 0.36 ^a^	1.16 ± 0.12 ^d^	30.00 ± 0.52 ^a^	15.07 ± 0.51 ^ab^	3.85 ± 0.18 ^b^	8.68 ± 0.31 ^c^	2.15 ± 0.10 ^e^
Giza 111	1.06 ± 0.05 ^c^	37.34 ± 1.59 ^a^	7.53 ± 0.34 ^a^	Not detected	25.62 ± 0.89 ^e^	15.87 ± 0.27 ^a^	1.87 ± 0.19 ^d^	3.16 ± 0.16 ^f^	7.55 ± 0.25 ^b^
Crawford	1.26 ± 0.05 ^a^	26.44 ± 1.21 ^d^	6.62 ± 0.31 ^b^	6.04 ± 0.21 ^a^	28.79 ± 1.00 ^bc^	14.67 ± 0.25 ^b^	4.50 ± 0.16 ^a^	11.68 ± 0.20 ^a^	Not detected
Giza 82	0.88 ± 0.02 ^d^	28.12 ± 1. 09 ^cd^	4.36 ± 0.18 ^d^	5.25 ± 0.18 ^b^	29.74 ± 0.51 ^ab^	12.82 ± 0.44 ^c^	2.82 ± 0.13 ^c^	10.65 ± 0.37 ^b^	5.36 ± 0.13 ^d^
Giza 83	1.16 ± 0.03 ^b^	26.05 ± 0.91 ^d^	7.50 ± 0.34 ^a^	5.50 ± 0.25 ^b^	27.00 ± 0.47 ^d^	12.26 ± 0.54 ^c^	3.82 ± 0.13 ^b^	8.24 ± 0.35 ^d^	8.47 ± 0.29 ^a^
Holliday	1.33 ± 0.05 ^a^	29.50 ± 1.02 ^bc^	5.75 ± 0.26 ^c^	4.94 ± 0.22 ^c^	27.70 ± 0.48 ^cd^	14.41 ± 0.58 ^b^	3.62 ± 0.20 ^b^	7.10 ± 0.39 ^e^	5.65 ± 0.12 ^d^
*p*-value	<0.001	<0.001	<0.001	<0.001	<0.001	<0.001	<0.001	<0.001	<0.001

All data are expressed as the mean (*n* = 3) with ±SD in the same column; the a, b, c, d and e letters indicate significant differences between means (*p* < 0.01).

**Table 4:** DPPH radical scavenging activity IC_50_ (µg/mL) of soybean cultivar methanolic extracts.

Samples	DPPH Radical Scavenging Activity IC_50_ (µg/mL)
Giza 21	57 ± 2.01 ^d^
Giza 22	64 ± 2.43 ^c^
Giza 35	45 ± 1.56 ^f^
Giza 111	52 ± 1.80 ^e^
Crawford	61 ± 1.06 ^c^
Giza 82	81 ± 1.40 ^a^
Giza 83	55 ± 1.91 ^de^
Holliday	76 ± 2.23 ^b^
TBHQ	62 ± 1.07 ^c^
*p*-value	<0.001

All data are expressed as the mean (*n* = 3) with ±SD in the same column; the a, b, c, d, e and f letters indicate significant differences between means (*p* < 0.01).

**Table 5:** The antioxidant activity of soybean cultivars methanolic extract at 800 µg/mL determined as FRAP.

Cultivars	Ferric Reducing Power (µ Mole Fe^+2^/g Extract)
Giza 21	800 ± 40.41 ^d^
Giza 22	900 ± 40.57 ^d^
Giza 35	1900 ± 86.22 ^a^
Giza 111	1400 ± 66.58 ^b^
Crawford	1100 ± 51.32 ^c^
Giza 82	1200 ± 56.86 ^c^
Giza 83	1600 ± 72.11 ^b^
Holliday	770 ± 35.12 ^d^
TBHQ	2100 ± 111 ^a^
*p*-value	<0.001

All data are expressed as the mean (*n* = 3) with ±SD in the same column; the a, b, c and d letters indicate significant differences between means (*p* < 0.01).

**Table 6:** The percentage of HepG-2 viability as affected by soybean methanolic extracts at different concentrations (12.5, 25, 50, 100, and 200 µg/mL) from eight soybean cultivars compared to that of the control (Doxorubicin).

Cultivar	Concentration (µg/mL)/Cell Viability (%)				
	12.5	25	50	100	200
Giza 21	81 ± 2.81 ^a^	79 ± 2.74 ^ab^	68 ± 2.91 ^f–i^	59 ± 2.04 ^h–j^	45 ± 2.12 ^mn^
Giza 22	79 ± 2.74 ^ab^	76 ± 1.32 ^a–d^	62 ± 1.07 ^f–i^	55 ± 2.15 ^lm^	40 ± 1.39 ^no^
Giza 35	71 ± 2.92 ^b–e^	61 ± 2.11 ^g–i^	45 ± 1.78 ^mn^	35 ± 1.21 ^op^	10 ± 1.17 ^r^
Giza 111	76 ± 2.63 ^a–d^	68 ± 3.12 ^d^^–g^	51 ± 1.88 ^j–m^	44 ± 1.76 ^mn^	30 ± 1.04 ^p^
Crawford	79 ± 2.62 ^ab^	72 ± 1.99 ^b–e^	58 ± 2.01 ^h–k^	51 ± 1.77 ^j–m^	40 ± 2.77 ^no^
Giza 82	78 ± 1.70 ^a–c^	70 ± 2.42 ^c–f^	55 ± 0.95 ^i–l^	49 ± 2.39 ^lm^	38 ± 1.32 ^n^^–p^
Giza 83	75 ± 2.60 ^a–d^	66 ± 1.14 ^e–h^	49 ± 0.85 ^lm^	40 ± 1.83 ^no^	21 ± 1.36 ^q^
Holliday	90 ± 1.73 ^k–m^	80 ± 0.78 ^mn^	71 ± 2.46 ^b–e^	60 ± 1.04 ^g–i^	50 ± 2.29 ^k–m^
Doxorubicin	50 ± 1.15 ^j–m^	45 ± 0.78 ^mn^	35 ± 1.60 ^op^	20 ± 0.92 ^q^	9 ± 1.31 ^r^
ANOVA	*p*-value				
Cultivar	<0.001				
Concentration	<0.001				
Cultivar × Concentration	<0.001				

All data are expressed as the mean (*n* = 3) with ±SD in the same column; the a–r letters indicate significant differences between means (*p* < 0.01).

**Table 7:** The percentage of MCF-7 viability as affected by soybean methanolic extracts at different concentrations (12.5, 25, 50, 100, and 200 µg/mL) from eight soybean cultivars compared to that of the control (Doxorubicin).

Cultivar	Concentration (µg/mL)/ Cell Viability (%)				
	12.5	25	50	100	200
Giza 21	88 ± 2.10 ^b^	86 ± 1.49 ^bc^	75 ± 1.30 ^f–h^	66 ± 2.29 ^ij^	50 ± 2.37 ^n^^–q^
Giza 22	86 ± 1.94 ^bc^	83 ± 1.44 ^b–e^	69 ± 1.20 ^h–j^	62 ± 2.15 ^j–l^	47 ± 2.15 ^p^^–r^
Giza 35	78 ± 2.27 ^d–g^	68 ± 2.36 ^h–j^	50 ± 1.87 *^n^*^–q^	42 ± 1.45 ^rs^	20 ± 1.35 ^uv^
Giza 111	83 ± 1.80 ^b–e^	75 ± 2.60 ^f–h^	58 ± 2.01 ^k–m^	49 ± 1.70 ^o–r^	36 ± 1.25 ^s^
Crawford	86 ± 2.18 ^bc^	79 ± 2.62 ^c–g^	65 ± 2.98 ^jk^	58 ± 2.26 ^k–m^	47 ± 2.15 ^p^^–r^
Giza 82	85 ± 2.30 ^b–d^	77 ± 1.33 ^e–g^	62 ± 1.07 ^j–l^	52 ± 2.60 ^m–p^	44 ± 0.76 ^qr^
Giza 83	82 ± 1.42 ^b–f^	73 ± 1.26 ^g–i^	56 ± 0.97 ^l–o^	47 ± 2.15 ^p^^–r^	28 ± 1.28 ^t^
Holliday	97 ± 1.68 ^a^	87 ± 1.51 ^b^	78 ± 2.50 ^d–g^	67 ± 2.32 ^ij^	58 ± 1.00 ^k–m^
Doxorubicin	57 ± 0.99 ^l–n^	52 ± 1.80 ^m–p^	42 ± 1.45 ^rs^	27 ± 1.47 ^tu^	16 ± 0.73 ^v^
ANOVA	*p*-value				
Cultivar	<0.001				
Concentration	<0.001				
Cultivar × Concentration	<0.001				

All data are expressed as the mean (*n* = 3) with ±SD in the same column; the a–v letters indicate significant differences between means (*p* < 0.01).

**Table 8:** Origin and pedigree for the used eight soybean cultivars.

No.	Accession	Origin	Pedigree
1	Giza 21	Egypt	Crawford × Celest
2	Giza 22	Egypt	Crawford × Forrst
3	Giza 35	Egypt	Crawford × Celest
4	Giza 111	Egypt	Crawford × Celest
5	Crawford	USA	Williams × Colombus
6	Giza 82	Egypt	Crawford × M. Presto
7	Giza 83	Egypt	Selected from MBB-133-9
8	Holliday	USA	N77-179 × Johnston

## Data Availability

Not applicable.

## Sample Availability

Samples of the compounds … are available from the authors.

## References

[1] Fratianni F., Ombra M.N., Cozzolino A., Riccardi R., Spigno P., Tremonte P., Coppola R., Nazzaro F. (2016). Phenolic constituents, antioxidant, antimicrobial and anti-proliferative activities of different endemic Italian varieties of garlic (*Allium sativum* L.). J. Funct. Foods.

[2] Del Rio D., Costa L., Lean M., Crozier A. (2010). Polyphenols and health: What Compounds Are Involved?. Nutr. Metab. Cardiovasc. Dis..

[3] Chandrasekara A., Shahidi F. (2011). Bioactivities and antiradical properties of millet grains and hulls. J. Agric. Food Chem..

[4] Magalhaes S.C., Taveira M., Cabrita A.R., Fonseca A.J., Valentão P., Andrade P.B. (2017). European marketable grain legume seeds: Further Insight into Phenolic Compounds Profiles. Food Chem..

[5] Król-Grzymała A., Amarowicz R. (2020). Phenolic compounds of soybean seeds from two European countries and their antioxidant properties. Molecules.

[6] Amarowicz R., Shahidi F. (2017). Antioxidant activity of broad bean seed extract and its phenolic composition. J. Funct. Foods.

[7] Aguilera Y., Estrella I., Benitez V., Esteban R.M., Martín-Cabrejas M.A. (2011). Bioactive phenolic compounds and functional properties of dehydrated bean flours. Food Res. Int..

[8] Abdel-Shafi S., Al-Mohammadi A.-R., Sitohy M., Mosa B., Ismaiel A., Enan G., Osman A. (2019). Antimicrobial activity and chemical constitution of the crude, phenolic-rich extracts of Hibiscus sabdariffa, Brassica oleracea and Beta vulgaris. Molecules.

[9] Singh B., Singh J.P., Kaur A., Singh N. (2017). Phenolic composition and antioxidant potential of grain legume seeds: A Review. Food Res. Int..

[10] Afifi S. (2017). Isoflavones, Phenolics Content and Antioxidant Activity of Three Egyptian Soybean Cultivars. Al-Azhar J. Pharm. Sci..

[11] Abdel-Sattar E., Abdel-Monem A.R., Hegazy M.E.F., El-Halawany A.M., Afifi S.M. (2021). Genetic diversity, LC-ESI-MS chemical profile and in vivo antitumor activity of three Egyptian soybean cultivars. Nat. Prod. Res..

[12] Malenčić D., Popović M., Miladinović J. (2007). Phenolic content and antioxidant properties of soybean (*Glycine max* (L.) Merr.) seeds. Molecules.

[13] Lien D.T.P., Tram P.T.B., Toan H.T. (2015). Effects of extraction process on phenolic content and antioxidant activity of soybean. J. Food Nutr. Sci..

[14] Yin L., Zhang Y., Wu H., Wang Z., Dai Y., Zhou J., Liu X., Dong M., Xia X. (2020). Improvement of the phenolic content, antioxidant activity, and nutritional quality of tofu fermented with Actinomucor elegans. LWT-Food Science and Technology.

[15] Singleton V.L., Orthofer R., Lamuela-Raventós R.M. (1999). Analysis of total phenols and other oxidation substrates and antioxidants by means of folin-ciocalteu reagent. Methods in Enzymology.

[16] Jayaprakasha G.K., Patil B.S. (2007). In vitro evaluation of the antioxidant activities in fruit extracts from citron and blood orange. Food Chem..

[17] Riedl K.M., Lee J.H., Renita M., St Martin S.K., Schwartz S.J., Vodovotz Y. (2007). Isoflavone profiles, phenol content, and antioxidant activity of soybean seeds as influenced by cultivar and growing location in Ohio. J. Sci. Food Agric..

[18] Sakthivelu G., Akitha Devi M., Giridhar P., Rajasekaran T., Ravishankar G., Nikolova M., Angelov G., Todorova R., Kosturkova G. (2008). Isoflavone composition, phenol content, and antioxidant activity of soybean seeds from India and Bulgaria. J. Agric. Food Chem..

[19] Devi M.A., Gondi M., Sakthivelu G., Giridhar P., Rajasekaran T., Ravishankar G. (2009). Functional attributes of soybean seeds and products, with reference to isoflavone content and antioxidant activity. Food Chem..

[20] Kim S.-H., Song H.-K., Ahn J.-K., Kim J.-T., Hahn J.-S., Chung I.-M. (2004). Changes of phenol compounds according to storing years in soybean. Korean J. Crop. Sci..

[21] Annaji M., Poudel I., Boddu S.H., Arnold R.D., Tiwari A.K., Babu R.J. (2021). Resveratrol-loaded nanomedicines for cancer applications. Cancer Rep..

[22] Jia H., Yang Q., Wang T., Cao Y., Jiang Q.-Y., Sun H.-W., Hou M.-X., Yang Y.-P., Feng F. (2016). Rhamnetin induces sensitization of hepatocellular carcinoma cells to a small molecular kinase inhibitor or chemotherapeutic agents. Biochim. Biophys. Acta (BBA) Gen. Subj..

[23] Zhu G., Liu X., Li H., Yan Y., Hong X., Lin Z. (2018). Retracted: Kaempferol inhibits proliferation, migration, and invasion of liver cancer HepG2 cells by down-regulation of microRNA-21. Int. J. Immunopathol. Pharmacol..

[24] Li Y., Jiang F., Chen L., Yang Y., Cao S., Ye Y., Wang X., Mu J., Li Z., Li L. (2015). Blockage of TGFβ-SMAD2 by demethylation-activated miR-148a is involved in caffeic acid-induced inhibition of cancer stem cell-like properties in vitro and in vivo. FEBS Open Bio.

[25] Ponte L.G.S., Pavan I.C.B., Mancini M.C.S., da Silva L.G.S., Morelli A.P., Severino M.B., Bezerra R.M.N., Simabuco F.M. (2021). The hallmarks of flavonoids in cancer. Molecules.

[26] Kasprzak A., Kwasniewski W., Adamek A., Gozdzicka-Jozefiak A. (2017). Insulin-like growth factor (IGF) axis in cancerogenesis. Mutat. Res./Rev. Mutat. Res..

[27] Lin Y.-L., Liu Y.-K., Tsai N.-M., Hsieh J.-H., Chen C.-H., Lin C.-M., Liao K.-W. (2012). A Lipo-PEG-PEI complex for encapsulating curcumin that enhances its antitumor effects on curcumin-sensitive and curcumin-resistance cells. Nanomed. Nanotechnol. Biol. Med..

[28] Zare M., Norouzi Roshan Z., Assadpour E., Jafari S.M. (2021). Improving the cancer prevention/treatment role of carotenoids through various nano-delivery systems. Crit. Rev. Food Sci. Nutr..

[29] AOAC (1990). Official methods of Analysis. Aoac.

[30] Markham R. (1942). A steam distillation apparatus suitable for micro-Kjeldahl analysis. Biochem. J..

[31] Omar A., Al-khalaifah H.S., Mohamed W., Gharib H., Osman A., Algabri N.A., Amer S.A. (2020). Effects of Phenolic-Rich Onion (*Allium cepa* L.) Extract On the Growth Performance, Behavior, Intestinal Histology, Amino Acid Digestibility, Antioxidant Activity, and the Immune Status of Broiler Chickens. Front. Vet. Sci..

[32] Ordonez A., Gomez J., Vattuone M. (2006). Antioxidant activities of Sechium edule (Jacq.) Swartz extracts. Food Chem..

[33] Ramadan M.F., Osman A.M.O., El-Akad H.M. (2008). Total antioxidant potential of juices and beverages-Screening by DPPH in vitro assay. Wiss. Verlagsgesellschaft. Stuttg..

[34] Re R., Pellegrini N., Proteggente A., Pannala A., Yang M., Rice-Evans C. (1999). Antioxidant activity applying an improved ABTS radical cation decolorization assay. Free Radic. Biol. Med..

[35] Hansen M.B., Nielsen S.E., Berg K. (1989). Re-examination and further development of a precise and rapid dye method for measuring cell growth/cell kill. J. Immunol. Methods.

